# Supplementary material for: Development of a Quality-of-Life Instrument to Measure Current Health Outcomes: Health-Related Quality of Life with Six Domains (HRQ-6D)
Source: J Clin Med. 2023 Apr 11;12(8):2816. doi: 10.3390/jcm12082816 (PMC10140924; doi:10.3390/jcm12082816)
Supplement: Supplementary file 1 [file jcm-12-02816-s001.zip › jcm-2223948-supple.pdf]

Appendix 1

Health-related Quality of Life with Six Dimensions (HRQ-6D)
Kualiti Hidup Berhubung Kesihatan dengan Enam Dimensi

Instruction:

The aim of this questionnaire is to measure how you feel about your quality of life related to health conditions for TODAY. For each question, please choose the most appropriate option from Strongly disagree to Strongly agree as the answer that best describes your situation. Please read the questions carefully and mark (x) your best options.

Arahan:

Soal selidik ini bertujuan untuk mengukur kualiti hidup anda daripada keadaan kesihatan untuk HARI INI. Untuk setiap soalan, sila pilih jawapan daripada pilihan Sangat tidak setuju kepada Sangat setuju sebagai jawapan yang paling hampir menggambarkan situasi yang anda alami. Sila baca setiap soalan dengan teliti dan tandakan (x) pilihan terbaik anda.

No.	Items / *Perkara*	Strongly disagree *Sangat tidak setuju*	Disagree *Tidak setuju*	Neutral *Neutral*	Agree *Setuju*	Strongly agree *Sangat setuju*
1	I feel pain at any part of my body *Saya rasa sakit di mana-mana bahagian badan saya*	( )	( )	( )	( )	( )
2	I feel unhealthy *Saya rasa tidak sihat*	( )	( )	( )	( )	( )
3	I feel lack of physical energy *Saya rasa kurang tenaga fizikal*	( )	( )	( )	( )	( )
4	I feel tired even at rest *Saya rasa letih walaupun dalam keadaan rehat*	( )	( )	( )	( )	( )
5	I feel depressed *Saya berasa tertekan*	( )	( )	( )	( )	( )

6	I feel anxious<br>*Saya berasa cemas*	( )	( )	( )	( )	( )
7	I have difficulty to move from one place to another<br>*Saya menghadapi kesukaran untuk bergerak dari satu tempat ke satu tempat yang lain*	( )	( )	( )	( )	( )
8	My movements are slower than people of my age<br>*Pergerakan saya lebih lambat daripada orang yang seusia dengan saya*	( )	( )	( )	( )	( )
9	I have problem attending to my self-care needs<br>*Saya menghadapi masalah untuk mengurus keperluan diri sendiri*	( )	( )	( )	( )	( )
10	I have problem doing household chores<br>*Saya menghadapi masalah untuk melakukan kerja-kerja rumah*	( )	( )	( )	( )	( )
11	I am worried that I will suffer poor health within 5 years<br>*Saya bimbang saya akan menghadapi kesihatan yang semakin teruk dalam masa 5 tahun*	( )	( )	( )	( )	( )
12	I am worried that my lifespan is shorter than people of my age<br>*Saya bimbang jangka hayat kehidupan saya lebih pendek daripada orang seusia saya*	( )	( )	( )	( )	( )

A scoring mechanism for HRQ-6D
Mekanisme pemarkahan untuk HRQ-6D

Major domain	Domains	Item	Scoring mechanism
Health	1.Pain	Q1,Q2	[(Q1 + Q2)/10] x 100
	2.Physical Energy	Q3,Q4	[(Q3 + Q4)/10] x 100
	3.Emotion	Q5,Q6	[(Q5 + Q6)/10] x 100
		Q1 until Q6	[(Q1+Q2+….+Q6)/30] x 100
Body function	4.Mobility	Q7,Q8	[(Q7 + Q8)/10] x 100
	5.Self-care	Q9,Q10	[(Q9 + Q10)/10] x 100
		Q7 until Q10	[(Q7+Q8+….+Q10)/20] x 100
Perception	6.Perception of future health	Q11,Q12	[(Q11 + Q12)/10] x 100
Total		Q1 until Q12	[(Q1+Q2+….+Q12)/60] x 100

Question on measuring health status

1. Status of health (choose and mark ONLY one answer)
   Status kesihatan (pilih dan tanda SATU jawapan sahaja)
   ( ) I am healthy (never been diagnosed with any medical condition except mild fever or headache, and I have never been hospitalised except for child delivery).
   Saya sihat (tidak pernah disahkan dengan mana-mana penyakit kecuali demam atau pening kepala, dan saya tidak pernah dimasukkan ke hospital kecuali untuk melahirkan anak)
   ( ) I have been diagnosed with one or more than one diseases but never been hospitalized (except for child delivery).
   Saya telah disahkan menghidap satu atau lebih daripada satu penyakit tertentu tetapi tidak pernah dimasukkan ke dalam hospital (kecuali untuk melahirkan anak)
   ( ) I have been diagnosed with one or more than one diseases and have been hospitalized due to disease progression or complications.
   Saya telah disahkan menghidap satu atau lebih daripada satu penyakit tertentu dan telah dimasukkan ke dalam hospital kerana penyakit berkenaan semakin parah atau disebabkan komplikasi penyakit
   ( ) I have been diagnosed with one or more than one diseases and have been hospitalized more than 3 times due to disease progression or complications.
   Saya telah disahkan menghidap satu atau lebih daripada satu penyakit tertentu dan telah dimasukkan ke dalam hospital lebih daripada 3 kali kerana penyakit berkenaan semakin parah atau disebabkan komplikasi penyakit
   ( ) I am dependent on medicine and/or medical procedure(s) and/or medical equipment to keep me alive (e.g. major surgery, dialysis for end stage renal disease, blood transfusion for thalassemia, heart transplant/stenting for heart problem, chemotherapy for cancer, etc.)
   Saya sedang bergantung kepada ubat dan/atau prosedur perubatan dan/atau peralatan perubatan untuk meneruskan kelangsungan hidup (cth: pembedahan besar, dialysis, pemindahan darah untuk thalasemia, pemindahan jantung, penggunaan sten untuk masalah jantung, kimoterapi untuk kanser dan/atau lain-lain.)

2. Kindly describe your **HEALTH** quality of life **ONLY** in the last **TWO** weeks based on this scale by circling any number from 0 (poorest health quality of life) to 10 (excellent health quality of life). Health quality of life refers to one's satisfaction of his/her quality of life based on his/her health condition only.
   *Sila gambarkan kualiti hidup anda dari aspek **KESIHATAN SAHAJA** dalam **DUA** minggu yang lepas berdasarkan skala ini dengan bulatkan mana-mana nombor daripada "0" (kualiti hidup yang sangat teruk) sehingga "10" (kualiti hidup yang sangat baik).*

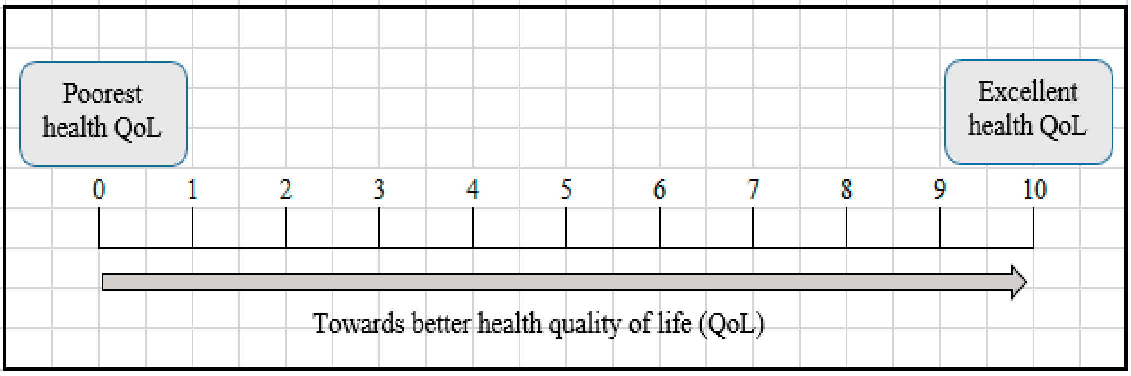

End of questions
